# Quantification and biodegradability assessment of meso-2,3-dimercaptosuccinic acid adsorbed on iron oxide nanoparticles†

**DOI:** 10.1039/c9na00236g

**Published:** 2019-08-12

**Authors:** S. Bemowsky, A. Rother, W. Willmann, J. Köser, M. Markiewicz, R. Dringen, S. Stolte

**Affiliations:** UFT – Centre for Environmental Research and Sustainable Technology, Department Sustainable Chemistry, University of Bremen Leobener Straße 6 D-28359 Bremen Germany; CBIB – Centre for Biomolecular Interactions Bremen, Neurobiochemistry, Faculty 2 (Biology/Chemistry), University of Bremen Leobener Straße 5/NW2 D-28359 Bremen Germany; UFT – Centre for Environmental Research and Sustainable Technology, Department Neurobiochemistry, University of Bremen Leobener Straße 6 D-28359 Bremen Germany; Technische Universität Dresden, Faculty of Environmental Sciences, Department of Hydrosciences, Institute of Water Chemistry Bergstraße 66 01069 Dresden Germany stefan.stolte@tu-dresden.de

## Abstract

Many interesting applications of magnetic iron oxide nanoparticles (IONPs) have recently been developed based on their magnetic properties and promising catalytic activity. Depending on their intended use, such nanoparticles (NPs) are frequently functionalized with proteins, polymers, or other organic molecules such as meso-2,3-dimercaptosuccinic acid (DMSA) to improve their colloidal stability or biocompatibility. Although the coating strongly affects the colloidal properties and environmental behaviour of NPs, quantitative analysis of the coating is often neglected. To address this issue, we established an ion chromatographic method for the quantitative analysis of surface-bound sulfur-containing molecules such as DMSA. The method determines the amount of sulfate generated by complete oxidation of sulfur present in the molecule. Quantification of the DMSA content of DMSA-coated IONPs showed that reproducibly approximately 38% of the DMSA used in the synthesis was adsorbed on the IONPs. Tests for the biodegradability of free and NP-bound DMSA using a microbial community from a wastewater treatment plant showed that both free and NP-bound DMSA was degraded to negligible extent, suggesting long-term environmental stability of DMSA-coated IONPs.

## Abstract 2

An analytical method was developed to quantify the amount of free and nanoparticle-bound dimercaptosuccinic acid to address the question whether or not the coating material is biodegradable.

## Graphical abstract

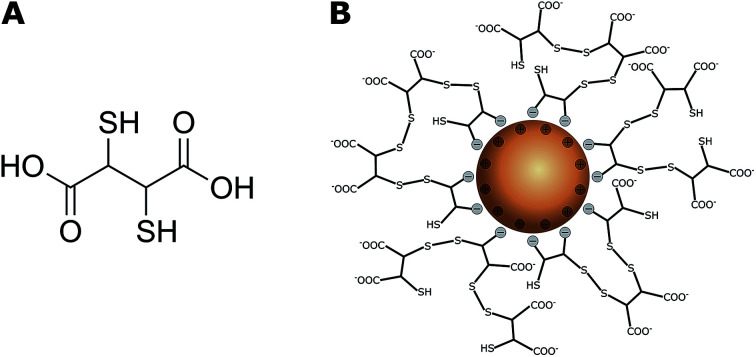

## 1. Introduction

**Fig. 1:**
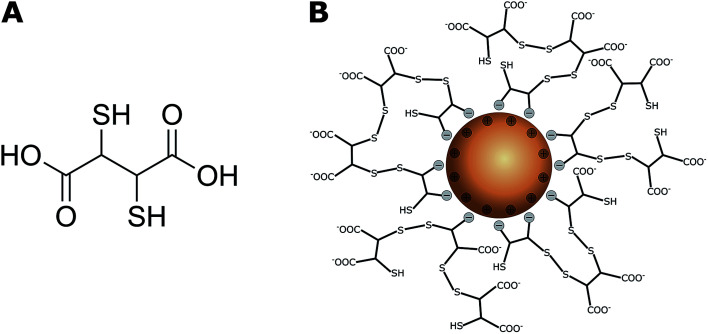
(A) Chemical structure of meso-2,3-dimercaptosuccinic acid (DMSA); (B) DMSA-IONP: functionalized surface *via* binding of DMSA carboxylate groups and cage formation by disulfide bridges.^32^ The thiol and negatively charged carboxylate groups on the surface can effectively capture heavy metal ions.^41^

Magnetic iron oxide nanoparticles (IONPs) with either magnetite (Fe_3_O_4_) or maghemite/hematite (Fe_2_O_3_) as the cores have received much attention in recent decades because of their unique magnetic properties and high catalytic activities.^1,2^ They have been developed for a wide range of biomedical and environmental applications. These include biomedical imaging, cellular labelling and tracking in tumour therapy, targeted drug delivery systems,^3,4^ and soil and groundwater remediation,^5^ including wastewater treatment.^6,7^ Their use in remediation is based on their efficient removal of toxic metal ions from aqueous solutions *via* adsorption,^7,8^ after which they can be removed by applying external magnetic fields.^9^ Hu *et al.* reported adsorption capacities of 17.0–19.2 mg g^−1^ for Cr(vi), 26.8 mg g^−1^ for Cu(ii), and 23.6 mg g^−1^ for Ni(ii) by maghemite nanoparticles (NPs).^10,11^ Uncoated IONPs have high surface energies because of their large surface-to-volume ratios and are therefore prone to particle agglomeration,^6^ and this tends to minimize surface energies. NP-specific properties such as superparamagnetism,^12^ reactivity, and mobility^6^ can consequently change. Furthermore, they are sensitive to air oxidation^13^ because of their high chemical activities. IONPs are therefore often coated with inorganic or organic compounds such as Au,^14,15^ silica,^16^ humic acid,^17^ and dextran^18,19^ or other polymers^20,21^ to enhance their colloidal stability and reduce the probability of magnetite IONP oxidation in aqueous media.^22^ A coating can also be used to endow IONPs with specific surface functionalities for cell labelling and targeting,^3^ or ion binding to increase metal ion adsorption for water treatment processes.^6^ In particular, NPs functionalized with meso-2,3-dimercaptosuccinic acid (DMSA, Fig. 1A) have potential applications in drug delivery systems,^23,24^ as heating agents for magnetic hyperthermia^25–27^ and remediation of heavy-metal-contaminated environments.^28,29^

DMSA contains two sulfhydryl groups (–SH),^30,31^ and oxidation of DMSA molecules forms a cage *via* disulfide crosslinking around the IONPs and modifies the surface charges of the particles because of an excess of carboxylic groups (Fig. 1B).^32^

Because DMSA as a thiol-containing compound is a good chelator of metals such as arsenic, cadmium, lead, and mercury^31,33,34^ and is categorized as not harmful to humans or other mammals, it is approved for clinical chelation therapy.^35–37^ Similarly, studies of DMSA-coated IONPs (DIONPs) have shown weak or no genotoxicity and cytotoxicity towards mammalian cells such as cultured brain astrocytes^38^ and human dermal fibroblasts^39^ at the highest tested concentrations, namely 0.22 and 0.10 g Fe L^−1^ (4000 and 1791 μM iron). However, uncoated IONPs affected brain nerve cells of mice *via* induction of oxidative stress and morphological damage on administration of 130 μg of IONPs.^40^ Few studies have evaluated the environmental effects of DIONPs and uncoated IONPs.^41–43^ The consequences of exposing aquatic organisms such as duckweed (*Lemna minor*), green algae (*Raphidocelis subcapitata*), or water fleas (*Daphnia magna*) to such NPs have recently been investigated.^41^ The determined EC_50_ values (72 h) for green algae range from 0.05 mg Fe L^−1^ for uncoated IONPs to 0.13 mg Fe L^−1^ for DIONPs (0.86–2.27 μM Fe). DIONPs showed no effect and uncoated IONPs showed moderate toxic effects on *Daphnia* (EC_50_ 21–66 mg Fe L^−1^ and 374–1181 μM Fe, respectively) after 72 h, but ingestion and accumulation of coated and uncoated IONPs was observed in the gastrointestinal tract of daphnids. Neither type of IONPs (with or without DMSA coating) affected *Lemna*; this was because of particle agglomeration in the medium. The DMSA coating apparently mitigates the ecotoxicity of IONPs and therefore environmental processes which cause degradation of the coating and release of IONPs will essentially increase the environmental hazard posed by these NPs. In the aforementioned studies, comprehensive particle characterization was performed by atomic absorption spectroscopy, atomic force spectroscopy, dynamic and electrophoretic light scattering (DLS/ELS), flow field-flow fractionation, transmission electron microscopy, and other techniques.^41,44–46^ However, information on the amount of DMSA adsorbed on NP surfaces is not yet available. In particular, the ratio of bound/free DMSA and its changes over time (*e.g.* as a result of biodegradation) are important in environmental hazard assessment because the stability, biological effects, and transport behaviours of DIONPs in any environmental medium will be directly affected.

The standard electrochemical procedures such as capillary or gel electrophoresis which are used for the detection and separation of NPs by size and shape^47^ cannot be used to determine and quantify surface-adsorbed biomolecule concentrations. In electrochemical analysis, NPs in the sample can cause problems such as adsorption on the electrode surface^47^ or can affect the signal sensitivity of UV/Vis detectors,^48^ which are often used in electrochemical methods. Known methods of detection for DMSA include high-performance liquid chromatography (HPLC)^30,34,49^ paired with fluorescence detection or a gas chromatographic system coupled with a flame ionization detector (GC-FID).^50^ DMSA tends to oligomerize in atmospheric oxygen because of formation of disulfide bridges. Quantification of DMSA usually requires conversion of oligomers to monomeric DMSA *via* chemical reactions, *e.g.* with dithiothreitol, or electrochemically. Limits of quantification for GC-FID and HPLC are 150 μmol L^−1^ and 0.14–0.55 μmol L^−1^ (depending on physiological matrices) respectively. These methods are not suitable for NPs or solutions with high metal ion concentrations, because of lack of volatility, the possibility of capillary blocking, or interference with detection. To avoid DMSA oligomerization in analytical samples, we suggest complete oxidation of the thiol groups of DMSA to sulfate ions in the presence of *aqua regia* at high temperatures. The sulfate concentration can be assessed quantitatively by ion chromatography (IC). In this study, this analytical approach was validated and used to determine the ratio of bound/free DMSA.

Chemical transformations of nanomaterials in the environment include adsorption of macromolecules, biodegradation of coatings, dissolution, oxidation, reduction, and sulfidation, many of which have been studied in detail, but less attention has been paid to transformations of surface coatings.^51,52^ In particular, the effects of aqueous photochemical reactions, biodegradation, and similar processes on adsorbed macromolecules lead to corona changes and strongly affect surface interactions and reactivity, which in turn affect the transport, fate, and toxicity of NPs.^52–54^ It has been shown that poly(ethylene oxide) which was covalently linked to NPs was biologically degraded by bacteria enriched from urban river water.^55^ The microbial degradation of some macromolecules can occur extracellularly by secreted enzymes,^56,57^ but the degradation of water-soluble molecules by bacteria often requires uptake into the cell.^56,58^ Intracellular degradation of the coating of Au NPs by enzymes in rats^59^ and embryonic mouse fibroblasts^60^ was detected for proteins (bovine serum albumin) and poly(isobutylene-*alt*-maleic anhydride) with a dodecyl side chain, respectively. Further examples of transformations of adsorbed biomolecules include photochemical degradation of the citrate coating of CeO_2_ NPs^61^ or the thiolated poly(ethylene glycol) corona of Au NPs.^54^

For coated NPs, in most cases it is not possible to comment on the amount of adsorbed material. Because the concentrations are unknown, assumptions have to be made for interpretation of the experimental data, as described by Zhang *et al.*^41^ After the release of DIONPs into the environment, microbial degradation of the surface coating can lead to a change in particle behaviour. It is therefore important to determine how much substance is adsorbed on the NP surfaces. In this study, we established and validated a quantitative method for the determination of NP-bound DMSA and then examined whether or not it was biodegradable. To determine the amount of DMSA coated on DIONP, the NPs were digested and the thiol groups of the surface-adsorbed DMSA were oxidized to sulfate, which was then quantified by IC.

## 2. Materials and methods

### 2.1 Materials

All chemicals were purchased in the highest purity available from Sigma-Aldrich (Steinheim, Germany), Merck (Darmstadt, Germany), or VWR (Langenfeld, Germany). This includes DMSA (≥97.5%), FeCl_3_ (≥99.99%), HCl (≥37%, puriss p.a.), HNO_3_ (≥65%, puriss p.a.), H_2_SO_4_ (95–97%, puriss p.a.), K_2_SO_4_ (≥99%), KI (≥99.5%, puriss p.a.), Na_2_CO_3_ (99.95–100.05%), and NaHCO_3_ (≥99.7%). EPA-certified (United States Environmental Protection Agency) thread vials (20 mL, clear glass, ND24) from neo-Lab (Heidelberg, Germany) were used for colloidal stability testing. Twenty-four-well microtitre plates were obtained from Sarstedt (Nümbrecht, Germany). MilliQ water (resistivity 18.2 MΩ cm) was produced with a Millipore MilliQ Plus water purification system (Burlington, MA, USA).

### 2.2 Procedures for synthesis of DIONPs

#### 2.2.1 IONP synthesis

Magnetic IONPs (γ-Fe_2_O_3_) were synthesized according to a previously described method.^62,63^ A solution (380 mL) containing FeCl_3_·6H_2_O (8.89 g, 32.9 mM), FeCl_2_·4H_2_O (3.28 g, 16.5 mM), and 37% HCl (1 mL) was thoroughly mixed. Slow addition of 25% (w/v) NH_4_OH solution (25 mL) induced precipitation under vigorous stirring. The resulting black magnetic precipitate was collected, isolated with a permanent magnet (NdFeB-magnet, Webcraft, Uster, Switzerland), and washed twice with deionized water (100 mL). The precipitate was heated with 2 M HNO_3_ (40 mL) until the colour of the mixture changed to dark brown. The product was magnetically collected, separated from the supernatant, and then heated with 0.34 M Fe(NO_3_)_3_·9H_2_O (60 mL) at 90 °C for 30 min according to Bee *et al.*^62^ The IONPs were magnetically separated from the supernatant, dispersed in deionized water to a final volume of 50 mL, and then filtered sterile through a 0.2 μm filter (syringe filter, cellulose acetate membrane of pore size 0.2 μm, Sigma-Aldrich, Steinheim, Germany).

#### 2.2.2 DIONP synthesis

The synthesized IONPs were coated with DMSA after determination of the iron content (see Section 2.2.3). DMSA (0.13 g, 0.7 mmol) was dissolved at 50 °C in double-distilled water (150 mL) under stirring. This solution was added to 40 mM iron (100 mL) in the form of IONPs under vigorous stirring resulting in a final concentration of DMSA equal to 2.85 mmol L^−1^ (*c*_initial_). After mixing for 30 min at room temperature, the particulate content was separated by centrifugation at 800 rcf for 5 min. Due to the DMSA coating of the IONP, the solution becomes acidic, which is why the NPs agglomerate. Therefore, it is easier to separate them *via* centrifugation and to remove the supernatant, whereby almost no excess DMSA from synthesis should be present. The particles were resuspended in double-distilled water (80 mL) and the pH of the dispersion was adjusted to 10 with NaOH and then to 7.4 with HCl. By adjusting the pH to 10, the NP surfaces are charged more negatively, which leads to increased repulsion and better dispersion of the NPs. After adjusting to pH 7, the DIONPs were stable for several months. The resulting dispersion was filtered through a 0.2 μm filter. The final iron concentration of the DIONP dispersion was 2.31 ± 0.25 g Fe L^−1^ (41.31 ± 4.45 mM Fe).

#### 2.2.3 Determination of iron content

The iron content was determined by a modified version of a previously reported colourimetric ferrozine-based method.^63^ The synthesized IONP dispersion (10 μL) was mixed with 37% HCl (40 μL), followed by dilution with 50 mM NaOH (950 μL). The solution (100 μL) was then mixed with 10 mM HCl to a volume of 200 μL. The sample was mixed with freshly prepared iron-releasing reagent (100 μL; 1 : 1 mixture of 1.4 M HCl and 4.5% w/v KMnO_4_ in double-distilled water) and then fresh iron-detection reagent (30 μL; 2.5 M ammonium acetate, 1 M ascorbate, 6.5 mM ferrozine, and 6.5 mM neocuproine) was added.^64^ After reaction for 30 min at room temperature, samples (280 μL) were placed in the wells of a microtitre plate (Sarstedt, Nümbrecht, Germany) and the absorbance of the iron–ferrozine complex at 540 nm was recorded with a Sunrise RC microtitre plate photometer (Tecan, Crailsheim, Germany). The iron content was determined by comparing the absorbance of the sample to those of defined iron standard solutions (FeCl_3_ in 10 mM HCl).

### 2.3 Method validation

To investigate the amount of DMSA adsorbed on NPs, the NPs were digested, and the organic coating was oxidized to enable determination of the thiol groups of DMSA as sulfate. An IC method was established with K_2_SO_4_ as the standard for the concentration range 5–95 μM. To calculate the amount of DMSA bound to the particles two types of analytical samples were prepared (for details please see Fig. S1 in the ESI†): ‘sample A’ after coating, centrifugation, washing and filtration through 0.2 μm syringe filter contained NP-bound DMSA and free DMSA that coprecipitated with the pellet (*m*_pellet_ = *m*_free_ + *m*_bound_); ‘sample B’ after additional step of ultrafiltration contained only free DMSA that was present in the pellet (*m*_free_). The ultrafiltration was performed using Vivaspin 500 centrifugal filters (Sartorius, Göttingen, Germany) with a polyethersulfone membrane (3000 molecular-weight cut-off). The filter was rinsed four times with deionized water (500 μL) at 14 100 rcf for 30 min prior to centrifuging the NPs to avoid contamination with interfering ions in the IC analysis. The amount of bound DMSA was then calculated as: *m*_bound_ = *m*_pellet_ − *m*_free_.

Release of iron ions during digestion of the DIONP samples could affect the analysis, therefore additional iron chloride (10 μL of 50 mM FeCl_3_, 0.125 mM in the sample) was added to the calibration standards prior to their treatment to investigate its influence on the analysis.

#### 2.3.1 Quantification of DIONP bound DMSA

Four independent DIONP batches (A–D) were investigated, which were synthesized and coated using an identical protocol as mentioned in 2.2.2. The DIONP stock solutions of about 40 mM Fe were diluted by a factor of 40 to approximately 1 mM Fe. These diluted suspensions were further used for a concentration series (6 concentrations between 0.10–0.52 mM Fe in 3 replicates each) to determine the amount of DMSA adsorbed to DIONPs. After initially washing the pellet of coated DIONPs still contained some amount of free DMSA. To account for that, we have also measured this amount by removing coated NPs through ultrafiltration and putting the permeate through digestion protocol.

Both types of samples (A and B) were digested with *aqua regia* at high temperature to oxidize DMSA to water-soluble sulfate and to dissolve the NPs (in sample A). An aqueous sample (100 μL) consisting of DIONPs (sample A) or free DMSA (sample B) was mixed with 37% HCl (80 μL) and ≤69% HNO_3_ (20 μL), followed by addition of 50 mM FeCl_3_ (10 μL). The mixture was homogenized for 30 s with a vortex mixer (Heidolph, Schwabach, Germany) and then centrifuged in a Minispin Plus centrifuge (Eppendorf, Hamburg, Germany) for 30 s at 6700 rcf. The organic material in the sample was oxidized overnight at 95 °C in a thermoblock (Eppendorf, Thermomixer compact). The dried sample was then dissolved in the IC eluent (1 mL; mixture of Na_2_CO_3_ and NaHCO_3_). To avoid transfer of residual particles in sample A into the IC system, 700 μL of this sample were transferred to a new cup and centrifuged once more for 30 min at 14 100 rcf. The 500 μL of both samples were vortexed and centrifuged again at 14 100 rcf for 30 min.

Finally, the 500 μL of each sample were diluted with eluent (1480 μL) and an internal anion standard (20 μL; 5 mM KI), homogenized for 20 s with a vortex, and then analysed *via* IC. For samples without FeCl_3_ the last centrifugation step was omitted and 500 μL of the sample were directly diluted with eluent and internal standard. For a flowchart of sample preparation please see Fig. S1 in ESI file.†

#### 2.3.2 Dynamic and electrophoretic light scattering (DLS/ELS)

For testing the biodegradability, the coated NPs must be brought into suspension. In this context the colloidal stability of the DIONPs was tested for a period of 28 days; this corresponds to the duration of the performed biodegradation test. Analysis was performed with a Delsa™ Nano C particle analyser (Beckman Coulter, Krefeld, Germany) with either a cuvette cell for measurement of hydrodynamic diameters or a flow cell for zeta potential measurements. The device featured a diode laser (30 mW, *λ*_0_ = 658 nm), with scattered light detection by a photomultiplier tube and analysis with a digital correlator. DLS experiments were performed at a backscattering angle of 165°. After shaking the samples manually for 10 s to ensure sampling of homogenously distributed particles, a sample (2.5 mL) was placed in a MilliQ-water-cleaned polystyrene cuvette (Sarstedt, Nümbrecht, Germany) of dimensions 10 × 10 × 45 mm^3^. This sample was then thermostatted at 25 °C for 5 min in the analytical device before 10 repetitions with 120 s measurement times. The correlation function *g*^2^ was evaluated on the basis of the properties of pure water with a refractive index *n* (658 nm, 25 °C) = 1.3328 and a viscosity *η* (25 °C) = 0.8898 cP with Beckman Coulter Software Delsa™ Nano (version 3.730/2.30). For data interpretation, the cumulants method was used to calculate the *z*-average of the hydrodynamic diameter *d* and the polydispersity index.

The zeta potentials of the samples were determined by ELS under the same conditions as those used for DLS, except the scattering angle was changed to 15°. A sample (5 mL) was directly injected into a flow cell and equilibrated, after which three repetitions with a measurement time of 300 s were performed. Data were evaluated by the Smoluchowski equation from the refractive index, viscosity, and dielectric constant (*ε* = 78.3) of pure water with Beckman Coulter software (see DLS section). All measurements were recorded as three independent replicates for each sample.

#### 2.3.3 Ion chromatography (IC)

IC was used to indirectly quantify DMSA by determining the amount of sulfate formed by complete oxidation of the DMSA thiol groups. All chromatographic analyses were performed using a Metrohm Model 881 Compact IC system (Metrohm, Herisau, Switzerland) with a suppressor module and a column oven at 30 °C equipped with a Metrosep A Supp 5 column (150 × 170 mm) in combination with a Metrosep A Supp 4/6 Guard and a Metrosep RP 2 Guard column, an online degasser, and a 20 μL injection loop. Anions were detected using a conductivity detector (maintained at 30 °C) with an eluent mixture consisting of 3.2 mM Na_2_CO_3_ and 1.0 mM NaHCO_3_ at a flow rate of 0.7 mL min^−1^. Diluted H_2_SO_4_ was used for regeneration of the IC suppressor module (cation exchanger). Prior to preparation of the eluent mixture and regenerating solution, the deionized water was filtered through a 0.45 μm filter and degassed. All data were recorded with Metrohm software MagICNet (version 2.4 compact) and evaluated based on the peak area and signal of the iodide internal standard, 50 μM KI, which was used to correct for peak area variations over time.

Calibration was performed with K_2_SO_4_ over the concentration range 5–95 μM in concentration steps of 10 μM. Five independent repetitions were performed for each sample. The detection limit was determined with the student *t*-test for a confidence interval of 95% and the quantification limit corresponded to three times the detection limit.

### 2.4 Biodegradation test

A manometric respirometry method, namely OECD 301 F (Organisation for Economic Cooperation and Development, OECD, 2006) was used to measure the pressure decrease in test vessels caused by consumption of oxygen used by bacteria to degrade the sample.^65^ The test mixture (final volume 432 mL) contained a mineral medium (8.5 mg L^−1^ KH_2_PO_4_, 21.75 mg L^−1^ K_2_HPO_4_, 22.13 mg L^−1^ Na_2_HPO_4_·2H_2_O, 1.7 mg L^−1^ NH_4_Cl, 27.5 mg L^−1^ CaCl_2_, 22.5 mg L^−1^ MgSO_4_·7H_2_O, and 0.25 mg L^−1^ FeCl_3_), microbial inoculum, nitrification inhibitor (allylthiourea, 5 mg L^−1^), and 20 mg L^−1^ of the test substance. The microbial inoculum was derived from activated sludge from an aeration tank at the municipal wastewater treatment plant in Delmenhorst, Germany, with an average dry sludge content of 5 g L^−1^. Prior to the experiments, flocs were allowed to settle and then discarded. The remaining supernatant was aerated for 7 days and used as the inoculum after addition of a medium containing 10^4^ to 10^5^ colony-forming units per millilitre on average, determined by a Paddle test (Hach Lange, Düsseldorf, Germany). The test substances were weighed and combined with 1 L of inoculum in volumetric flasks. Two samples of volume 432 mL were taken from each flask and placed in amber-glass test bottles (OxiTop, WTW). Because of the limited sensitivity of the technique (lowest measurable range 40 mg O_2_ L^−1^) the concentrations of the test substance were significantly higher than expected environmental concentrations. Each sample test was run in duplicate, with blank samples to account for endogenous cellular breathing, and positive controls containing benzoic acid in the same concentration as that of the sample substance (20 mg L^−1^). During the test, the temperature was maintained at 20 °C. The decrease in pressure inside the bottle caused by oxygen consumption was measured, recorded, and converted to the biochemical oxygen demand (BOD). Finally, the percentage degradation was calculated from the BOD value and theoretical oxygen demand of DMSA, according to the guidelines.^65^

## 3. Results and discussion

### 3.1 Calibration of sulfate and effect of digestion process

**Fig. 2:**
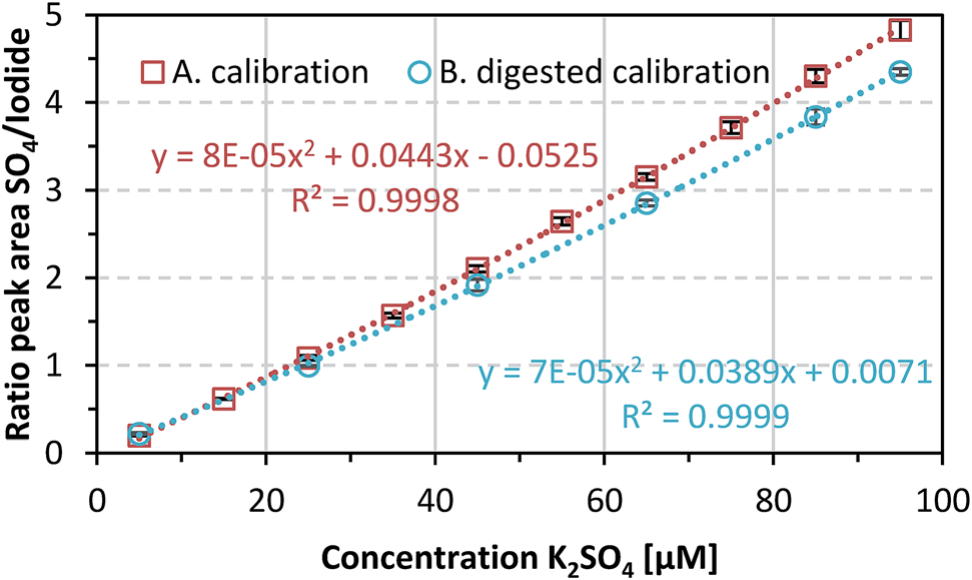
(Curve A) Calibration (*n* = 5) of K_2_SO_4_ over the concentration range. (Curve B) Calibration and determination of recovery rates of K_2_SO_4_ subjected to the same digestion procedure as the samples. Data represent mean values ± SD (*n* = 3).

The data shown in Fig. 2 curve A represent mean values of K_2_SO_4_ standards (5–95 μM) with standard deviations (*n* = 5). This calibration curve showed that the sulfate limit of detection (LOD) was 0.32 mg L^−1^ (1.83 μM) and the limit of quantification (LOQ) was 0.96 mg L^−1^ (5.49 μM). An internal anion standard, 50 μM KI, was used to correct for peak area variations (in the range 2.5–6.3%) over time from the quotient of the sulfate and iodine peak areas in the chromatograms. This ratio was used to obtain a calibration curve; a linearity check indicated second-order regression (*R*^2^ = 0.9998).

The sulfate recovery rates in the oxidation protocol were investigated for K_2_SO_4_ concentrations of 5, 25, 45, 65, 85, and 95 μM (Fig. 2 curve B). These samples showed average sulfate recovery rates of 93.7 ± 8.0% over three independent repetitions. A comparison of the two calibration curves showed that the area ratio after sample oxidation decreased by an average of 10.28 ± 1.21% over the concentration range, resulting in a higher LOD and LOQ of 0.57 mg L^−1^ (3.29 μM) and 1.72 mg L^−1^ (9.87 μM), respectively. A possible cause of the reduced signal and lower sensitivity could be loss of sulfate during sample preparation and treatment.

A direct comparison of the calibration curves (see Fig. S2 in the ESI†) obtained with and without addition of iron ions showed no significant difference (*p* = 0.4613 > 0.05). This indicates that the presence of iron ions did not affect the detection of sulfate and the added FeCl_3_ was not measurably contaminated with sulfate during production. The recovery rate was 98.5 ± 7.2%. However, the LOD and LOQ for the calibration curve with added FeCl_3_ decreased by approximately 44% to 0.14 mg L^−1^ (0.81 μM) and 0.42 mg L^−1^ (2.43 μM), respectively. This enables better quantification of sulfate from the DMSA-containing samples. It seems that the presence of iron ions affects the sensitivity by reducing the measurement variability, resulting in lower LOD and LOQ values.

### 3.2 Quantification of DMSA

**Fig. 3:**
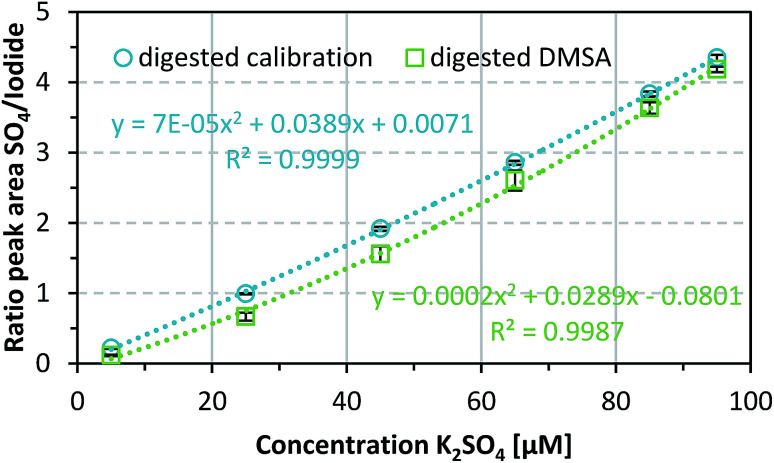
Influence of digestion process on sulfate recovery of DMSA in comparison to the calibration and determination of recovery rates of K_2_SO_4_ after the digestion procedure. Data represent mean values ± SD (*n* = 3).

After successfully verifying the reproducibility of the sulfate detection method, the method was used to determine known DMSA concentrations of 0.1, 0.5, 0.9, 1.3, 1.7, and 1.9 mM. DMSA contains two thiol groups, therefore two equivalents of sulfate are released by oxidation of one equivalent DMSA. If this fact and dilution through sample preparation are taken into account, the theoretical sulfate concentrations correspond to those of the calibration samples (5, 25, 45, 65, 85, and 95 μM). As shown in Fig. 3, an acceptable mean recovery rate for the digested DMSA samples, namely 80.6 ± 17.4% over three independent repetitions, was achieved. The sequence of steps needed for the sample preparation (see also ESI Fig. S1†) is one possible reason for the variations in the DMSA recovery rate. Another reason might have been an incomplete digestion of DMSA, therefore not all of the substance would be present as measurable sulfate. This is also true for surface adsorbed DMSA since a basic medium is used during the coating process and thiols tend to form disulfide bonds under these conditions which may affect the recovery rate of DMSA by incomplete oxidation to sulfate. Furthermore, the potential maximum recovery may be lower because the purity of DMSA is given as ≥97.5%, and on drying a maximum loss of 1% water can occur.

#### 3.2.1 Effect of dissolved iron on digestion

**Fig. 4:**
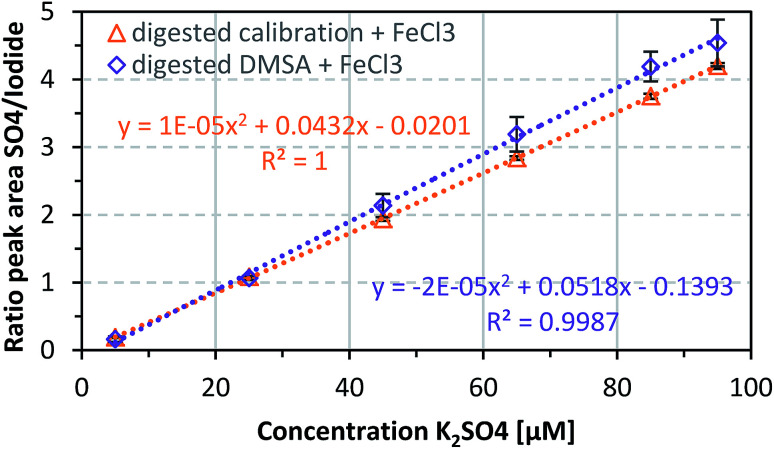
Influence of FeCl_3_ addition (0.125 mM in sample) on the DMSA digestion and recovery rate of sulfate. Data represent mean values ± SD (*n* = 3).

Treatment of IONPs with *aqua regia* results in complete release of iron ions from the NP core. To investigate the potential effect of iron ions on the DMSA quantification process, FeCl_3_ was added to samples of different DMSA concentrations. Fig. 4 shows that the addition of iron ions increased the sulfate recovery rate to about 100% (104.5 ± 10.3%) for selected concentrations in the range 5–95 μM.

Metal ions such as Fe^3+^ can be weakly bound by DMSA because of its chelating properties.^31,66,67^ This might reduce DMSA oligomerization, which enables more complete oxidation of sulfur and therefore higher sulfate recovery rates. Iron ions could also act as redox catalysts and have a positive effect on the oxidation of sulfur to sulfate.^68,69^ As a result, the concentrations of DMSA will be determined more precisely, with a higher recovery.

#### 3.2.2 Determination of the amount of DMSA in DIONP coating

The IC analysis (Table 1) showed that average 1.08 mmol DMSA was bound per litre of suspension containing on average 41.31 mmol iron (the molar ratio of DMSA to iron equal to 0.027). It also means that approximately 38% of DMSA used in the synthesis was bound onto the NPs. The reproducibility of NP synthesis was estimated by comparing the quantities of adsorbed DMSA coatings. The ratios of bound DMSA to total iron content for DIONPs in the four analysed batches (Table 1) did not differ significantly (*p* > 0.05). The relative DMSA coating quantities therefore indicate that the individual synthesis and coating processes gave highly reproducible coatings. Furthermore, the total surface area of the IONP can be estimated based on the particle diameter of 5–10 nm determined by TEM^70^ and the assumption of a spherical shape. Depending on the size distribution, an area of 116–232 m^2^ g^−1^ is estimated for the IONP, which corresponds to an adsorption of 0.27–0.53 mg DMSA m^−2^. It can be assumed that the IONP surface is almost completely saturated with DMSA. Higher concentrations of DMSA in synthesis provide comparable adsorbed amounts. This is important because the NP coating has a decisive effect on the environmental fate of the NPs and differences in the coating can lead to severe changes in NP behaviour.^71^

**Table 1:** Total, bound, and free DMSA concentrations of four separate DIONP batches as well as the ratio of bound DMSA to the concentration of iron. Data represent mean values ± SD (*n* = 18, 3 repetitions with 6 concentrations each)

Batch	*c* (Fe) [mmol L^−1^]	*c* (DMSA total) [mmol L^−1^]	*c* (DMSA bound) [mmol L^−1^]	*c* (DMSA free) [mmol L^−1^]	Molar ratio DMSA (bound)/Fe
A	46.13	1.35 ± 0.15	1.13 ± 0.14	0.22 ± 0.08	0.0245 ± 0.0029
B	40.49	1.24 ± 0.32	1.00 ± 0.34	0.24 ± 0.12	0.0247 ± 0.0084
C	43.00	1.25 ± 0.32	1.02 ± 0.34	0.23 ± 0.03	0.0237 ± 0.0079
D	35.60	1.36 ± 0.45	1.18 ± 0.42	0.19 ± 0.04	0.0331 ± 0.0118
Mean	41.31 ± 4.45	1.30 ± 0.31	1.08 ± 0.31	0.22 ± 0.07	0.0265 ± 0.0078

For the biodegradation tests, a known concentration of the biomolecule in the test system is required. Knowledge of the quantities of bound and free DMSA in the DIONP samples enabled us to investigate the biodegradability of the produced material.

In this regard, the IC analysis showed that samples used for biodegradation testing contained not only bound DMSA but also some dissolved DMSA oligomers amounting to approximately 20% of the bound amount (Table 1).

### 3.3 Characterization of DIONP

DLS measurements showed that after incubation for 28 days the DIONP were colloidally stable in water. The hydrodynamic diameter (*d*) was 39.1 ± 10.5 nm in diluted dispersions and showed no significant change over the time of the incubation period. In the biodegradation medium OECD 301 F the DIONP were not colloidally stable and agglomerated. The hydrodynamic diameter in this medium showed a slow and steady increase during the incubation period of 28 days from 41.3 ± 10.7 nm in the beginning (0 days) to the end (28 days) with 946 ± 252 nm (Tables 2 and 3). According to the Derjaguin–Landau–Verwey–Overbeek theory, the ionic strength strongly affects the surface charge and colloidal stability of NP dispersions.^72^ As the ionic strength of the OECD 301 F medium (21.5 mM) is much higher than that of MilliQ water, particle repulsion is weaker and the probability of contact between particles rises. Additionally, the medium contains Ca^2+^ and Mg^2+^ ions, which are known to promote particle–particle interactions by bridging effects, and thus agglomeration of NPs.^21,73^

**Table 2:** Hydrodynamic diameter *d*, polydispersity index PI, zeta potential *z* and pH-value of DIONP dispersed in MilliQ water (4.78 mM Fe as DIONP)

Time [d]	Parameter			
	*d* (distribution size) [nm]	PI	*z* [mV]	pH
0	43.6 ± 11.6	0.225	−49.6 ± 5.69	8.14
1	46.3 ± 12.1	0.231	−47.0 ± 2.92	7.52
2	43.8 ± 12.4	0.226	−47.6 ± 6.49	7.90
7	42.8 ± 11.0	0.225	−39.9 ± 3.52	8.33
14	41.9 ± 11.0	0.237	−47.5 ± 2.76	7.73
21	38.3 ± 9.9	0.278	−43.8 ± 3.90	8.06
28	39.1 ± 10.5	0.286	−45.4 ± 3.75	7.44

**Table 3:** Hydrodynamic diameter *d*, polydispersity index PI, zeta potential *z* and pH-value of DIONP dispersed in OECD 301 F medium (4.78 mM Fe as DIONP)

Time [d]	Parameter			
	*d* (distribution size) [nm]	PI	*z* [mV]	pH
0	41.3 ± 10.7	0.214	−37.5 ± 1.47	7.72
1	73.3 ± 20.8	0.204	−37.0 ± 0.82	7.72
2	96.3 ± 26.5	0.184	−37.5 ± 1.38	7.73
7	334 ± 86.8	0.259	−35.6 ± 0.64	7.69
14	546 ± 148	0.275	−37.4 ± 0.63	7.62
21	631 ± 165	0.267	−37.7 ± 0.46	7.56
28	946 ± 252	0.322	−37.2 ± 0.39	7.52

Large errors in diameters determined by DLS can arise because the measurements are based on the intensity of light scattered by particles in solution. Consequently, when calculating the diameter, larger particles are weighted more strongly than smaller particles because the contribution of scattered light to the correlation function scales with *r*^6^ (radius to the power of six). Light-scattering techniques such as DLS cannot detect individual particles inside agglomerates. In these techniques, all agglomerates are therefore regarded as single particles, resulting in a larger calculated diameter.

### 3.4 Biodegradability

**Fig. 5:**
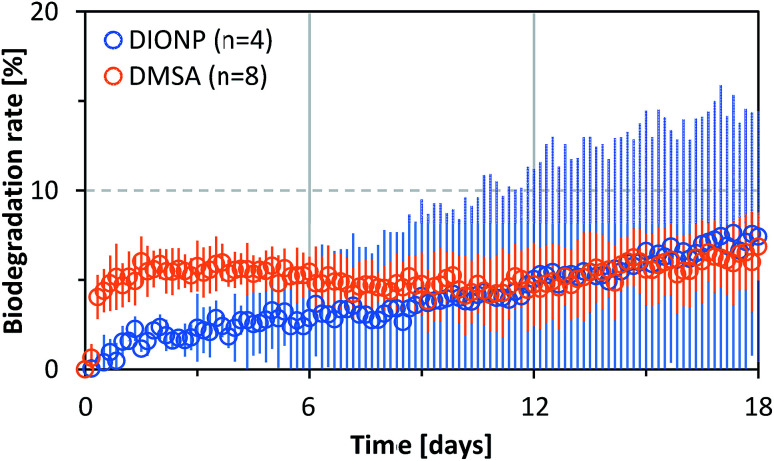
Biological degradation of DMSA in dissolved form (orange circle) or adsorbed on the surface of DIONP (blue circle). Data represent mean values ± SD (error range as an area of coloured lines, *n* = 4 for DIONP and *n* = 8 for DMSA).

We performed an ultimate biodegradability study with free DMSA and DIONPs. There was no significant degradation of DMSA in either case. As shown in Fig. 5, the degradation rates for free and bound DMSA were less than 10%. The test fulfilled the validity criteria (BOD in blanks, IONP blanks and degradation of benzoate within a given time frame; see Fig. S3 and S4 in ESI†). A control experiment with uncoated IONPs and benzoate showed that the NPs (or ions released from the NPs) had no effect on the biodegradability of an easily degradable substance, proving that the presence of NPs does not negatively affect the microbial community (see Fig. S4 in ESI†). A biodegradation of at least 60% initial BOD, which was achieved during the test period within a time frame of 10 days, can be considered as evidence of ready biodegradability in accordance with the OECD 301 F guidelines.^65^ The 10 days window starts when 10% biodegradation is reached. The 60% pass level represents virtually complete ultimate biodegradation of the test substance because it is assumed that the remaining 30–40% is assimilated by biomass or is present as products of biosynthesis.

The biodegradation was below 10% over the entire duration of the experiments, therefore the tested substances are considered to be not readily biodegradable; this contradicts the BIOWIN prediction. Quantitative structure–activity relationship models predict that the chemical structure of DMSA is readily biodegradable. The BIOWIN v.4.10, US Environmental Protection Agency Estimation Programs Interface Suite was used to predict the probability of biodegradation.^74^ Microbial conversion of mercaptosuccinic acid, which is structurally similar to DMSA, but has only one –SH functional group, has been reported in the literature.^75^ This supports the prediction made by the biodegradability model.

A key factor in the microbial degradation of biomolecules is the physical accessibility of the substance. For NPs or large biomolecules, the pathways for cellular entry into microorganisms are unfortunately insufficiently understood.^76^ In addition to the diffusion of small molecules through the membrane pores, endocytosis-like processes have been observed in which extracellular material is imported into the cells *via* the membrane transport system.^77–79^ A single DMSA molecule is smaller than 1 nm on the basis of sum of the known bond lengths (H–O, C–O, C–C) and could pass directly through membrane pores of size 4–50 nm.^80,81^ After uptake, small molecules can be biologically degraded by intracellular enzymes. DMSA tends to oligomerize by formation of disulfide bridges *via* thiol groups (–SH). This property of DMSA may strongly affect its bioavailability. The same applies to bound DMSA in the DIONP coating if microorganisms are unable to take up the coated NPs. For comparison we have predicted ultimate biodegradability of DMSA oligomers containing up to six DMSA units. Upon addition of the fifth DMSA molecule the applicability domain of the BIOWIN model in terms of molecular weight of the compound in question is exceeded. Despite that biodegradability actually increases with each additional DMSA unit.

Furthermore, the state of aggregation may vary in the range of environmental NP concentrations compared to the laboratory scale. NPs may be influenced by organic matter in aqueous systems, changing their behaviour by adsorbing to or exchanging the DMSA. The binding of strong ligands is of great importance for particle stability. One example is the interaction of the DMSA carboxylate groups on the IONP surface and the formation of a disulfide network.^32,82^ A more stable NP coating and thus reduced aggregation can influence particle properties such as stability and dissolution rates and change their bioavailability for pelagic organisms.^83^

The tendency of DMSA to form disulfide bridges and to polymerize probably complicates the uptake of DMSA from solution into microorganisms. Moreover, the DMSA coating on DIONPs may be less accessible to organisms which break down the coating from a free end group.^52^ The uptake of coated NPs into bacterial cells probably did not occur. The strong negative charge of NPs (Table 3) gives raise to repulsion between NPs and cells which are also negatively charged.^84^ Therefore, the lack of degradation of both DMSA oligomers in solution as well as DMSA present in NPs coating is most probably caused by their poor bioavailability.

## 4. Conclusions

We developed and validated a method for quantitative analysis of DMSA in IONP samples and used the method to determine the amounts of IONP-bound DMSA. It allowed us to determine the average amount of DMSA molecules present in the DIONPs coating with a quantification limit of 1.22 μmol L^−1^, which is about the same order of magnitude as the HPLC methods described in the literature, but with much simpler sample preparation. This method may be applicable to coated NPs of other metals and metal oxides if these NPs have a coating with a known sulfur content (as in the case of DMSA, which has two thiol groups) and these groups can be oxidized to sulfate. The method can therefore be used to verify the reproducibility of NP synthesis with regard to surface coating with DMSA or any other sulfur-containing coating.

DIONPs were colloidally stable in water over a long period of time but agglomerated in saline media because of the high ionic strength.

The stability of coating is an important issue defining fate of NPs in the environment.^85,86^ We have shown on a laboratory scale that the DMSA coating cannot be stripped of by the microbial community of wastewater treatment plant within the time range of the experiments. Under the selected aerobic test conditions, no significant degradation of DMSA was observed. In addition to the structure of the DMSA coating, the increasing size during incubation in the biodegradation medium may lead to reduced bioavailability. This suggests that the DIONP will be more stable than uncoated IONP in wastewater treatment plant, which is a likely release pathway considering possible application in medicine.^86,87^ The stability in freshwater will most probably be even higher due to lower salinity and less abundant microbial community.

These results suggest potential persistence of DMSA or DIONPs in the environment, but additional studies are needed to verify this first suspicion. Consequently, DIONPs could remain intact in wastewater treatment plants, albeit in agglomerated form, and interact with sewage sludge, for example.^88,89^ In some regions, sludge is used as fertilizer in agriculture and therefore enters other environmental compartments such as soils and sediment. During 2015, about 40% of the annual sewage sludge produced in Germany was recycled for agriculture and landscaping measures.^90^

Depending on the environmental conditions at the release site, coating of IONPs can affect particle behaviour, transport, or possible release of DMSA and iron ions. These effects also need to be investigated for potential long-term environmental exposures of NPs. While short-term exposures could cause only minor effects, extended exposure might lead to severe toxicity or inhibition of the organism metabolism.^91,92^ Especially low concentrations of NPs could develop effects after accumulation in the environment or organisms. The widespread use of nanotechnology in waste water treatment, agriculture and other sectors requires a more ecologically relevant system approach including long-term studies under environmental realistic scenarios.^93^

## Conflicts of interest

The authors declare no conflicts of interest.

## Supplementary Material

NA-001-C9NA00236G-s001
