# Supplementary material for: Quantification and biodegradability assessment of meso-2,3-dimercaptosuccinic acid adsorbed on iron oxide nanoparticles
Source: Nanoscale Adv. 2019 Aug 12;1(9):3670–9. doi: 10.1039/c9na00236g (PMC9419269; doi:10.1039/c9na00236g)
Supplement: NA-001-C9NA00236G-s001 [file NA-001-C9NA00236G-s001.pdf]

Please do not adjust margins

Nanoscale Advances

ARTICLE

Quantification and biodegradability assessment of meso-2,3-dimercaptosuccinic acid adsorbed on iron oxide nanoparticles

S. Bemowsky,[a] A. Rother,[a] W. Willmann,[b,c] J. Köser,[a] M. Markiewicz,[a,d] R. Dringen[b,c] and S. Stolte*[a,d]

Electronic Supplementary Information (ESI)

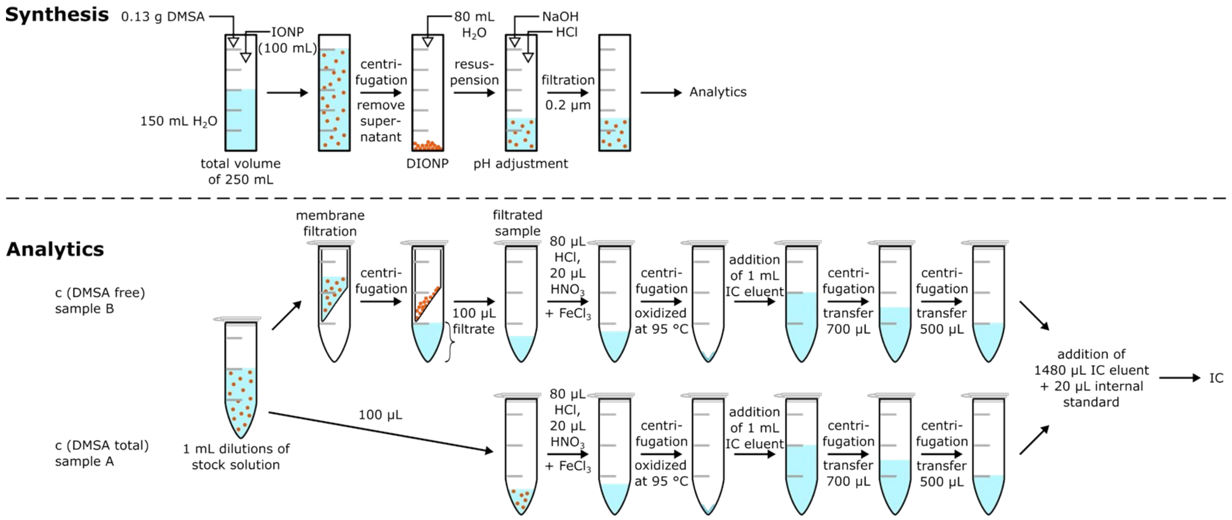

Fig. S1: Flowchart for sample preparation to determine bound DMSA concentrations.

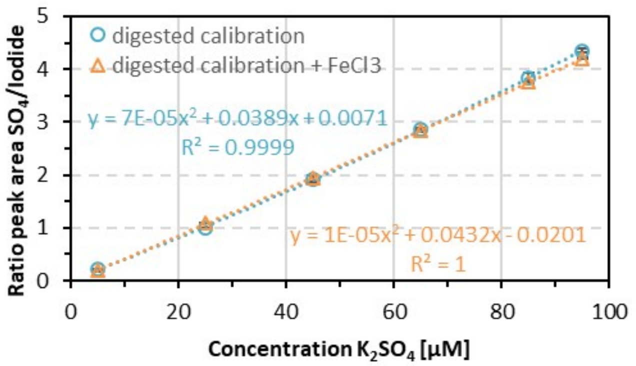

Fig. S2: Calibration and determination of recovery rates after the digestion process of K_2SO_4 over the concentration range from 5 to 95 µM with added $FeCl_3$ (0.125 mM). Data represent mean values ± SD (n = 3).

a. *UFT – Centre for Environmental Research and Sustainable Technology, Department Sustainable Chemistry, University of Bremen, Leobener Straße 6, D-28359 Bremen, Germany.*
b. *CBIB – Centre for Biomolecular Interactions Bremen, Neurobiochemistry, Faculty 2 (Biology/Chemistry) University of Bremen, Leobener Straße 5 / NW2, D-28359 Bremen, Germany.*
c. *UFT - Centre for Environmental Research and Sustainable Technology, Department Neurobiochemistry, University of Bremen, Leobener Straße 6, D-28359 Bremen, Germany.*
d. *Technische Universität Dresden, Faculty of Environmental Sciences, Department of Hydrosciences, Institute of Water Chemistry, Bergstraße 66, 01069 Dresden, Germany.*
**E-mail: stefan.stolte@tu-dresden.de*

Please do not adjust margins

Please do not adjust margins

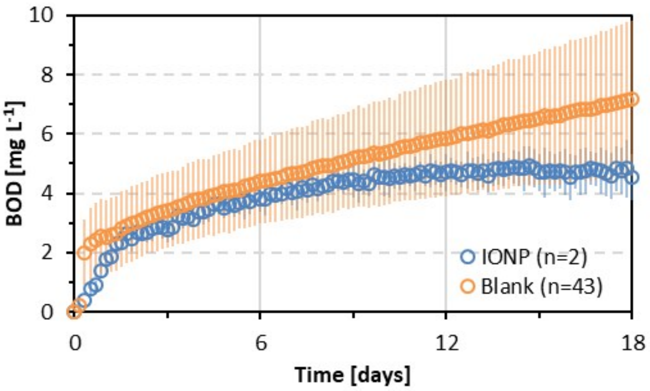

Fig. S3: Biochemical oxygen demand (BOD) of the sample controls (blank and uncoated iron oxide nanoparticles, IONP) for the biodegradation test system. Data represent mean values ± SD (error range as an area of coloured lines, n = 2 for IONP and n = 43 for blank).

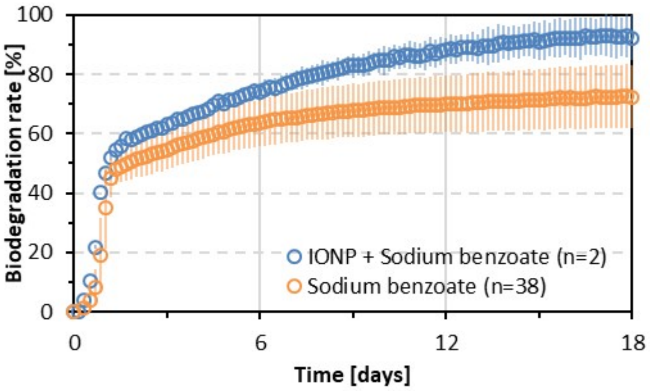

Fig. S4: Biological degradation of positive control sodium benzoate with and without the presence of IONP. Data represent mean values ± SD (error range as an area of coloured lines, n = 2 for IONP and n = 38 for sodium benzoate control).

This journal is © The Royal Society of Chemistry 2019

Please do not adjust margins
